# Perceptions of Climate Change Risk and Place Attachment: Parallel Indirect Associations Through In-Visit Evaluations of Memorable Tourism Experience and the Perceived Future Benefits of Edible Insects

**DOI:** 10.3390/insects17101086

**Published:** 2026-10-20

**Authors:** Yusuf Karakuş, Yasin Gökhan Kızıltan, Gökhan Onat

**Affiliations:** 1 Department of Tourism Management, Ardeşen Tourism Faculty, Recep Tayyip Erdoğan University, Rize 53400, Türkiye; yusuf.karakus@erdogan.edu.tr (Y.K.); yasin.kiziltan@erdogan.edu.tr (Y.G.K.); 2 Department of Gastronomy and Culinary Arts, Ardeşen Tourism Faculty, Recep Tayyip Erdoğan University, Rize 53400, Türkiye

**Keywords:** climate change risk perception, place attachment, memorable tourism experience, edible insects, Arabic-speaking tourists, Ayder Plateau

## Abstract

This study examines cross-sectional associations between climate change risk perception (CCRP) and place attachment (PA) among Arabic-speaking tourists visiting Ayder Plateau, Türkiye, through two parallel mediators: in-visit evaluations of memorable tourism experience qualities (MTE) and perceived future benefits of edible insects (PFBEI). Face-to-face convenience sampling between 22 July and 7 August 2026 yielded 435 questionnaires; 398 remained after multivariate outlier screening. Structural equation modeling and bias-corrected bootstrapping (5000 resamples) indicated positive associations of CCRP with MTE (β = 0.367) and PFBEI (β = 0.444), but no significant adjusted CCRP–PA association (β = 0.023). MTE (β = 0.699) and PFBEI (β = 0.165) were positively associated with PA, and both indirect associations were significant (MTE: β = 0.257; 95% CI [0.172; 0.353]; PFBEI: β = 0.073; 95% CI [0.034; 0.130]). With an unmeasured latent method factor, the MTE indirect association remained significant, whereas the PFBEI–PA association did not (β = 0.022; *p* = 0.750). MTE reflects momentary evaluations of the ongoing experience, not post-travel recall. Robustness to excluding 37 outliers could not be tested, country of origin was not recorded, and potential confounders were unmeasured; these are serious limitations, and the findings should be interpreted with caution.

## Simple Summary

Climate change concerns may be associated with how tourists evaluate the places they visit. This study surveyed 398 Arabic-speaking tourists during their visits to Ayder Plateau, a cool mountain destination in northeastern Türkiye. Respondents with higher climate risk perception reported more positive evaluations of the memorable qualities of their ongoing visit and stronger beliefs in the future benefits of edible insects. These experience evaluations had the strongest association with attachment to Ayder. The association between insect-related benefit expectations and attachment, however, became nonsignificant when a latent method factor was included. Insects were not offered or consumed as part of the destination experience examined here, so these results do not support an insect-based gastronomic explanation of attachment. The single-occasion survey cannot establish causation or whether the experiences remained memorable after travel. Respondents’ countries were not recorded, 37 excluded questionnaires could not be re-examined, and several potentially relevant variables were not measured, so the results should be interpreted with caution. The findings concern this convenience sample and suggest questions for future research on nature-based experiences, rather than demonstrating that climate concern or edible-insect beliefs strengthen destination attachment.

## 1. Introduction

Climate change is recognized as one of the global issues most profoundly affecting the ecological, economic, and social order of the twenty-first century. The Intergovernmental Panel on Climate Change’s latest synthesis report emphasizes that the effects of human-induced warming are now being observed in all regions of the world and that with each additional increase in warming, risks will become more complex and interdependent [1]. However, what drives individuals’ attitudes and behaviors toward climate change is not so much the objective risks themselves as how they are subjectively perceived. The literature on risk perception has long demonstrated that ordinary individuals assess dangers using emotional, intuitive, and sociocultural criteria, rather than expert calculations [2,3]. Consequently, the perception of climate change risk is garnering increasing attention as an explanatory psychological variable across a broad spectrum of behaviors, ranging from eco-friendly actions to food choices and travel decisions [4].

Tourism is one of the sectors with the most multifaceted relationship to climate change. Nature-based and mountainous destinations are particularly vulnerable to climate change, as their appeal is largely dependent on climatic conditions, unspoiled landscapes, and biodiversity. Current studies show that information and scenarios regarding climate change can alter visitors’ perceptions of a destination, their desire to visit, and their intention to return [5,6]. However, this literature has primarily focused on the effects of climate change on tourism demand and destination choice. The question of how general perceptions of climate risk relate to a visitor’s experience at a destination and the emotional and functional bonds they form with that place has received relatively little attention. In cool highland and mountain destinations, where climatic comfort is a decisive criterion in destination choice, understanding how visitors’ climate concerns are reflected in their experience and attachment processes is important from both theoretical and managerial perspectives.

In tourism literature, place attachment is one of the central constructs that explains visitors’ loyalty to a destination, their propensity to revisit, and their tendency toward environmentally friendly behavior [7]. Among the precursors of place attachment, the memorable tourism experience holds a special place; numerous studies have shown that positive, novel, and meaningful experiences at a destination facilitate the acquisition of personal meaning for the place and its integration into one’s identity [8,9,10,11]. In contrast, studies examining the relationship between perceived environmental risk and place attachment have mostly been conducted in contexts where the risk is directed directly at the place in question and where participants are residents of that place; these studies have shown that this relationship is neither one-sided nor automatic [12,13]. The question of whether a global and relatively abstract perception of climate threat among short-term visitors is directly linked to attachment to a specific destination, or whether this relationship is shaped through intermediary processes such as the destination experience, has not yet been sufficiently addressed.

The climate crisis is also raising new questions about the future of food systems. Given the high resource use and environmental impact of animal protein production, coupled with rising global protein demand, edible insects are emerging as a leading sustainable alternative protein source [14]. To date, the literature on alternative proteins has largely focused on individual acceptance and consumption preferences, as well as barriers to these preferences such as disgust, food neophobia, and cultural norms [15,16]. A recent study conducted in Turkey has shown that individuals who perceive climate risk as higher evaluate cultured meat and edible insects more positively [17]. However, regardless of individuals’ willingness to actually consume insects, the extent to which they believe these foods will contribute to addressing the global food crisis in the future has rarely been examined as a distinct construct. These macro-level benefit expectations, which align with the concept of response efficacy in climate psychology [4], are conceptually distinct from consumption intention. To the best of our knowledge, these expectations have not yet been examined within the same model for individuals engaged in tourism and experiencing a nature-based destination.

The context of this study is Ayder Plateau, located within the boundaries of Kaçkar Mountains National Park and known for its cool climate, lush greenery, and hot springs. The Eastern Black Sea Region has become an important summer destination for visitors from Gulf and Middle Eastern countries in recent years [18,19]. A local news report published shortly before fieldwork described reduced arrivals from several Middle Eastern markets in summer 2026 and attributed this to regional conflicts and airspace restrictions [20]. This contextual report does not quantify changes in the composition of the study sample. Although Arab tourists constitute a strategic market for regional tourism, research on perceptions of climate change and alternative foods has largely been based on Western and Asian samples. Recent studies conducted in Saudi Arabia, however, indicate that consumers in Saudi Arabia report significant reservations regarding edible insects, particularly concerning hygiene, sensory qualities, and halal compliance [21,22]. This visitor group provides a relevant context for examining climate-risk perceptions, food-benefit expectations, and attachment to a cool natural destination. However, Arabic-speaking visitors are heterogeneous, and edible-insect perceptions may differ across national and cultural groups. Because country of origin was not recorded in this study, such differences could not be examined within the sample.

Within this framework, the study addresses three questions. First, how is general climate risk perception associated with visitors’ current evaluations of their destination experience and place attachment? Second, are expectations regarding the future benefits of edible insects associated with attachment when experience evaluations are considered simultaneously? Third, how sensitive are these associations to a latent method factor and the exclusion of respondents with uniformly maximum scores after reverse coding? By examining an Arabic-speaking visitor sample at Ayder, the study extends the contextual coverage of this literature. It does not test cross-cultural differences or establish generalizability to Arab tourists as a whole.

The study’s contribution is to examine, within one cross-sectional model, two theoretically distinct routes linking general climate risk perception with attachment to a nature-based destination: a place-specific route through in-visit experience evaluations and a value-based route through expectations regarding the future benefits of edible insects. Edible insects are thus examined not as a destination food but as a sustainability-relevant belief that may make a protected natural place personally meaningful. The analysis also examines whether these associations persist under the reported sensitivity checks; the two pathways are not assumed to be equally robust. The distinction between in-visit evaluation and subsequent memory, the sensitivity of the PFBEI pathway to method variance, the untested robustness to outlier exclusion, the unrecorded country composition, and unmeasured covariates define the limits of the contribution.

The remainder of the study is organized as follows: Section 2 presents the theoretical framework of the constructs forming the research model, while Section 3 develops hypotheses based on the relevant literature. Section 4 describes the research methodology, while Section 5 presents the findings regarding measurements and the structural model. Section 6 discusses the findings in light of the literature; Section 7 presents conclusions and practical implications; and Section 8 evaluates the study’s limitations and offers recommendations for future research.

## 2. Theoretical Framework

This section defines what the four latent structures comprising the research model measure and what conceptual boundaries they encompass. Since cross-sectional survey data cannot definitively confirm the temporal sequence or causal direction among the variables, the following framework aims to theoretically justify the covariance patterns that the empirical data can test.

### 2.1. Perception of Climate Change Risk as a Subjective Assessment

Risk perception is not a direct replica of technical and statistical risk calculations in the individual mind. Slovic [2] demonstrated that ordinary people judge risks based on qualities distinct from expert assessments, such as emotional and intuitive dynamics (e.g., fear-inducing nature, uncertainty, lack of control, and potential for catastrophe), and that these judgments constitute an independent psychological reality that shapes behavior. Van der Linden [3] extends this approach to the field of climate change, arguing that climate risk perception is explained by the integration of cognitive (knowledge), experiential (emotions and personal experience), and sociocultural (norms, values) determinants, and that personal risk perception and societal risk perception can diverge from one another. In this study, Climate Change Risk Perception (CCRP) is, in this sense, a subjective belief and threat assessment; it does not indicate that participants were directly exposed to a climate-related hazard in the Ayder Plateau or that they possessed technical knowledge about the destination’s ecological vulnerability.

The nature of the measurement instrument also limits the conceptual interpretation. The scale items were adapted from a study conducted by Zobeidi et al. [23] with agricultural students; while in the original study participants were asked to assess the likelihood of certain outcomes occurring within the next twenty-five years due to global warming, in this study the same content was presented using a five-point Likert scale. Probability estimation and the degree of agreement are not cognitively identical; rather, the degree of agreement simultaneously reflects both conviction and concern regarding a potential outcome. Furthermore, with the removal of the item assessing personal health risk (CCRP5) from the measurement model based on psychometric and theoretical grounds, the structure included in the model primarily represents judgments of threats at the general, global, and societal levels. Therefore, the CCRP in this study should be interpreted primarily as a judgment regarding a general and distant climate threat. This distinction is central to understanding why the connection established with a specific micro-destination may not be directly linked to this general judgment.

### 2.2. Perceived Future Benefits of Edible Insects (PFBEI)

In the model, the construct termed “Perceived Future Benefits of Edible Insects” (PFBEI) is measured through four key statements: that consuming insects will be a cheaper way to meet protein demand; that insect consumption could solve global hunger in the future; that it will reduce greenhouse gas emissions from livestock; and that insects will serve as a viable alternative to traditional animal protein. These items do not measure whether individuals are willing to consume insects themselves, whether they have consumed them in the past, their level of food neophobia or aversion, their purchase intention, or the actual rate of adoption of insects in society. What is measured are beliefs regarding the expected future benefits and solution potential of edible insects in addressing the global food crisis.

The theory of innovation diffusion examines the adoption of an innovation among members of a social system over time through specific communication channels [24]. The items used in this study, however, rather than reflecting a temporal adoption process, capture expectations that a macro-level transformation will be effective, bearing a conceptual resemblance to “response efficacy” in climate psychology [4], without constituting a direct test of that construct. The items are based on an adaptation made by the researchers to insects, derived from Weinrich et al.’s [25] study on alternative protein sources (cultured meat). This is also why the framework is referred to as PFBEI rather than a name evoking the theory of innovation diffusion. Accordingly, throughout the study, the PFBEI framework is addressed at the level of optimistic benefit expectations regarding a sustainable food future in the face of the climate crisis, clearly distinguished from individual consumption acceptance or diffusion rate.

### 2.3. Memorable Tourism Experience Qualities Evaluated During the Visit

Kim et al. [8] conceptualized memorable tourism experiences in terms of experiences positively remembered after travel and developed a scale covering hedonism, refreshment, local culture, meaningfulness, knowledge, involvement, and novelty. The statements used in this study were adapted from the short form included in Zhang et al.’s [26] destination image model and encompass the uniqueness of the experience, impressions of local culture, enjoyable activities, and overall excitement. The five-item adaptation does not reproduce the full multidimensional scale.

The survey was administered during the trip, either during the visit or while respondents were resting at lodging facilities. Accordingly, MTE denotes in-visit evaluations of the qualities associated with a memorable tourism experience, not verified post-visit recall or enduring memorability. No follow-up assessment of memory was conducted. The prefix “As a responsible tourist…” was added to frame the items in the context of Ayder’s environmentally sensitive setting. This wording may also have primed environmental self-presentation and socially desirable responding, creating conceptual overlap with climate concern. The present design cannot separate such framing effects from substantive experience evaluations. Retaining the abbreviation MTE identifies the source construct; it does not establish equivalence between this adaptation and a retrospective MTE measure.

### 2.4. Place Attachment (PA) and Dimensionality

Scannell and Gifford [27] define place attachment within a tripartite framework comprising the dimensions of person (individual and collective meanings), psychological processes (emotion, cognition, behavior), and place (physical and social characteristics); they note that attachment is always directed toward a specific place with defined spatial boundaries. Brown and Raymond [28], on the other hand, address attachment in terms of two fundamental dimensions: place identity (the place being a part of the self, emotional and symbolic bonds) and place dependence (functional irreplaceability). In this study, place attachment (PA) has been adapted using Ayder Plateau as a reference to holistically measure the emotional and functional bond that tourists visiting Ayder Plateau establish directly with this destination. Thus, the space where the measured tourism experience takes place and the reference object toward which the attachment is directed have been made exactly equivalent, ensuring theoretical spatial alignment. Although it is known that these two dimensions can be distinguished in the tourism literature [7], in this study, the structure was modeled using a single-dimensional general index, guided by the principle of conceptual simplicity (and by comparing factor solutions).

### 2.5. An Integrative Rationale for the Two Pathways

Scannell and Gifford’s person–process–place framework [27] provides the organizing rationale for relating visitors’ psychological evaluations to attachment to a specified destination. Applied here, the person is the visitor, the place is Ayder Plateau, and the relevant psychological content comprises evaluations of the ongoing experience and the emotional and functional bonds expressed toward Ayder. CCRP supplies a general threat appraisal that may be associated with those evaluations, drawing on the risk-perception account of van der Linden [3]. This integration organizes the constructs but does not, by itself, establish their causal sequence or constitute a test of the complete tripartite framework.

The CCRP → MTE → PA pathway is the first of the two routes in the model. Experience and attachment share a destination-specific referent, and earlier research links positive tourism experiences with attachment [9,10]. The proposed positive CCRP–MTE association is more tentative: concern about environmental threats may coincide with heightened valuation of an ongoing nature-based visit. This is a possible interpretation to be examined, not an observed encoding process or evidence that climate concern caused the experience evaluation. Existing attachment may also influence experience ratings.

PFBEI concerns an anticipated response to food-system challenges at a global level; its conceptual connection with response efficacy [4] helps motivate the CCRP–PFBEI association. Its proposed association with attachment, which constitutes the second route, rests on value congruence rather than on a gastronomic experience at Ayder. According to value–belief–norm theory [29], beliefs about environmental problems and their solutions express underlying biospheric values, and the person dimension of place attachment comprises the values and meanings that individuals bring to a place [27]. Visitors who expect sustainable food innovations to help address the climate crisis may therefore find a protected, ecologically valued destination such as Kaçkar Mountains National Park congruent with those values and form stronger bonds with it, in line with evidence linking environmental orientations with attachment to natural and protected areas [7,30]. On this account, MTE and PFBEI represent two distinct routes: one through the evaluation of the destination experience and one through value-based expectations that make a sustainability-relevant natural place personally meaningful. Because insects were not offered or consumed at Ayder, the PFBEI pathway does not imply an insect-based gastronomic experience; evidence from an entomophagous community [31] concerns local consumption and is not used to support it. The underlying biospheric values were not measured directly, so value congruence is the theoretical rationale for H4 and H6 rather than a tested process, and general environmental orientation and common response tendencies remain possible alternative explanations. “Parallel” refers to simultaneous statistical estimation; the two routes are theoretically distinct, but the data do not demonstrate them as independent psychological processes.

## 3. Literature Review and Hypothesis Development

### 3.1. Perception of Climate Risk and Tourism Experience (H1)

In the tourism literature, climate change has mostly been examined in terms of destination demand and visit decisions. These studies show that information and scenarios regarding climate change can alter the way visitors evaluate nature-based destinations. For example, in a mountain ecotourism region in southern Mexico, certain climate scenarios reduced the desire to visit the destination [5], while among coastal tourists, knowledge of climate change was found to be associated with destination choice and the intention to revisit [6]. While these findings indicate that perceptions of climate risk are reflected in destination evaluations, they do not directly predict a positive relationship with experience evaluation. The rationale for H1 draws on two possible interpretations, neither of which is directly measured here. The first is the tendency, described in the “last-chance tourism” literature, for visitors to perceive natural areas, which are considered vulnerable due to climate change, as rarer and more valuable [32]. Visitors who perceive the climate threat as serious may therefore value the time they spend in a protected ecosystem, such as Kaçkar Mountains National Park, more highly. Second is the dimension of meaningfulness, one of the fundamental components of a memorable tourism experience [8]: environmental concern may be associated with more personally meaningful evaluations of natural elements. Memory encoding and destination-specific threat were not measured in this study. However, a reverse relationship is also theoretically possible; a positive nature-based experience may momentarily heighten environmental sensitivity and, consequently, the perception of risk. Since a cross-sectional design cannot distinguish between these two directions, H1 is stated as a correlational hypothesis.

**H1.**  *Perceived climate change risk is positively associated with in-visit evaluations of memorable tourism experience qualities.*

### 3.2. Climate Risk Perception and Expected Benefits of Edible Insects (H2)

The literature on alternative proteins indicates that environmental concerns may be positively associated with evaluations of novel foods. Systematic reviews have shown that acceptance is determined by taste, familiarity, disgust, neophobia, and norms [15,33]. Regarding the direct relationship between climate risk and alternative proteins, a recent study conducted in Turkey among respondents reported that individuals who perceive climate risk as higher evaluate cultured meat and edible insects more positively [17]. However, Onat et al. [16] found that the perception of future food insecurity is not directly related to the intention to consume insects but exhibits an indirect relationship mediated by neophobia. This study adds a new context to the literature by measuring macro-level benefit expectations (PFBEI) rather than individual consumption intentions among individuals within tourism mobility.

**H2.**  *Perceived climate change risk is positively associated with perceived future benefits of edible insects (PFBEI).*

### 3.3. Perceived Climate Risk and Place Attachment (H3)

The relationship between place attachment and environmental risk has generally been examined in resident samples where risk refers to that specific place, and this relationship is neither unidirectional nor automatic [12,13]. Extending a positive association to short-term visitors whose climate-risk judgments are global is therefore tentative. The CCRP → PA path is retained to estimate the association remaining after MTE and PFBEI are considered, rather than imposing a zero direct coefficient. Its inclusion permits assessment of the proposed positive association but does not imply that general climate concern must produce destination attachment.

**H3.**  *Perceived climate change risk is positively associated with place attachment.*

### 3.4. Expectations Regarding Edible Insects and Place Attachment (H4)

The literature on gastronomic tourism indicates that local food experiences can be associated with place attachment [9]. In the present setting, however, insect consumption was not part of the destination experience, so H4 does not rest on a gastronomic encounter. Instead, it follows from the value-congruence rationale outlined in Section 2.5: expectations that edible insects will help address the food and climate crisis reflect biospheric values [29], and visitors holding such values may form stronger bonds with a protected natural destination that is congruent with them [27,30]. Shared environmental orientation and response tendencies remain competing explanations of any observed association.

**H4.**  *Perceived future benefits of edible insects are positively associated with place attachment.*

### 3.5. Memorable Tourism Experience and Place Attachment (H5)

Tourism-experience research reports positive associations between MTE and place attachment. Studies of tourism experience and well-being [10] and of MTE among short-term visitors [11] support examining this relationship. Unique and culturally enriching experiences can acquire personal meaning in relation to a destination. Here, however, the hypothesis concerns visitors’ current evaluations of those qualities; it does not test whether lasting memories subsequently contribute to attachment.

**H5.**  *In-visit evaluations of memorable tourism experience qualities are positively associated with place attachment.*

### 3.6. Indirect Relationships (H6 and H7)

**Figure 1:**
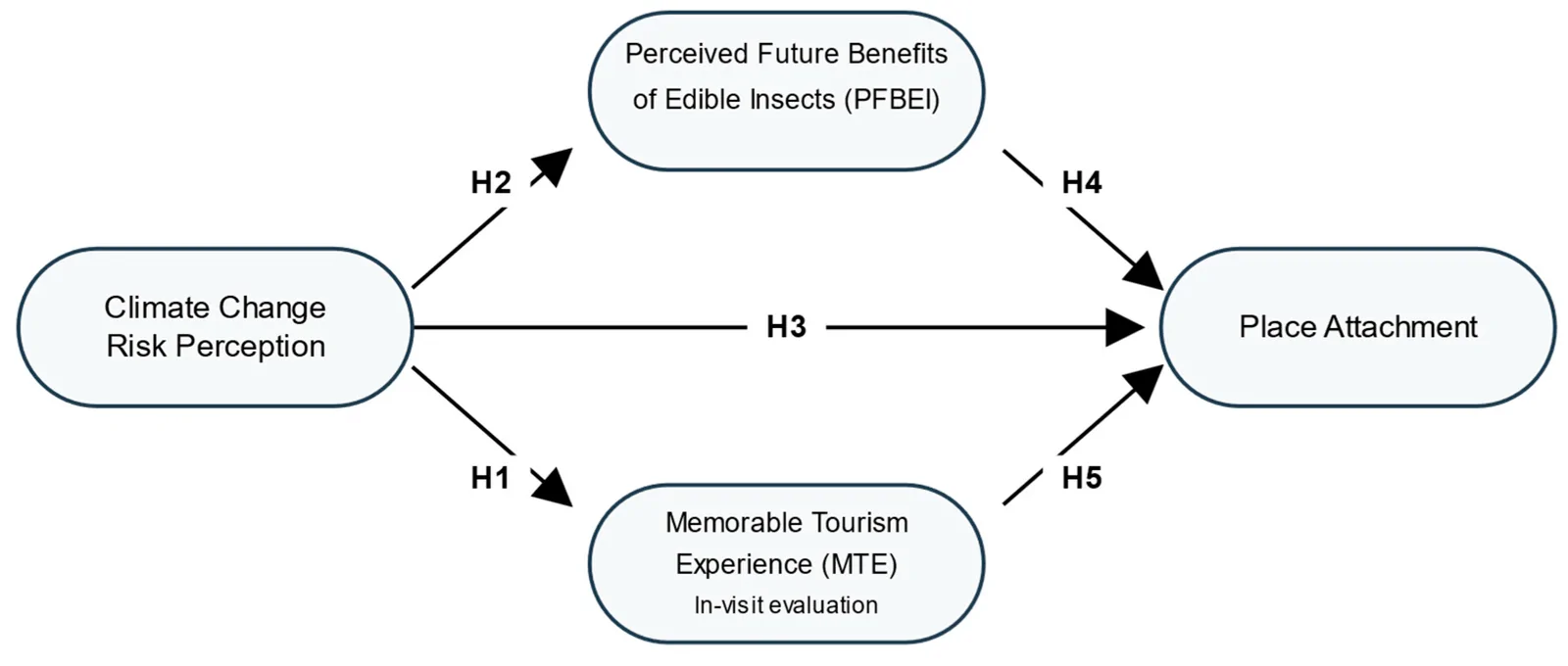
Research Model. Note: Arrows show the regression specification, not established causal direction. H6 and H7 represent the indirect associations through PFBEI and MTE, respectively. The PFBEI and MTE residual covariance is freely estimated in the statistical model and omitted from the diagram for clarity. CCRP: climate change risk perception; MTE: in-visit evaluations of memorable tourism experience qualities; PFBEI: perceived future benefits of edible insects; PA: place attachment.

The simultaneous model (Figure 1) decomposes the CCRP–PA association into a direct coefficient and specific indirect coefficients through MTE and PFBEI [34,35]. The place-specific experience rationale motivates H7, whereas H6 follows from the value-congruence rationale for H4 (Section 2.5). The statistical procedures for estimating indirect coefficients do not provide substantive evidence for either proposed pathway. Because the data are cross-sectional, these products of coefficients represent model-dependent indirect associations, not temporal or causal mediation [36].

**H6.**  *There is a positive indirect association between climate change risk perception and place attachment through the perceived future benefits of edible insects (PFBEI).*

**H7.**  *There is a positive indirect association between climate change risk perception and place attachment through in-visit evaluations of memorable tourism experience qualities (MTE).*

## 4. Method

### 4.1. Study Area and Sample

The study was conducted at Ayder Plateau, located within the boundaries of Çamlıhemşin District, Rize Province, in Turkey’s Eastern Black Sea Region. Ayder Plateau, one of the lowest-elevation entry points to Kaçkar Mountains National Park, is known not only for its rich flora and fauna but also for its healing hot springs. Designated as a Tourism Center in 1987, the region is a major attraction due to the diverse tourism activities it offers. In recent years, in particular, the share of visitors from Gulf and Middle Eastern countries in the region’s tourism has steadily increased; the Eastern Black Sea Region has become a strategic summer destination for Arab tourists due to its cool climate, lush natural beauty, and cultural offerings [18,19]. A local report described reduced arrivals from several Middle Eastern markets shortly before fieldwork [20]. This raises a possible seasonal-selection concern, but no comparative visitor-profile data were collected to establish how the sampled visitors differed from those in other seasons.

Recruitment targeted visitors whose native language is Arabic, who reside in Middle Eastern or Gulf countries, and who were in the region for vacation purposes. “Arabic-speaking tourists” is used as an operational description of this recruitment group, not as a verified nationality or culturally homogeneous category. However, country of origin and country of residence were not recorded in the questionnaire, so adherence to the residence criterion cannot be documented. This is a questionnaire design flaw. The national composition of the sample is therefore unknown, and possible cultural differences between national groups in edible-insect perceptions and place attachment could not be examined (Section 8). The primary reason for this selection is the significant share of the Middle Eastern market in destination tourism [18]. Second, the literature on alternative foods and the climate crisis has largely focused on the West and Asia; consequently, the sustainability perceptions of this strategic tourist group have not been adequately represented in the literature. While historical sources indicate that locust consumption existed in the past among some nomadic communities in the Arabian Peninsula [37], recent studies demonstrate that some consumers in Saudi Arabia report reservations to insects based on concerns regarding hygiene, sensory preferences, and halal compliance [21,22]. Therefore, the purpose of selecting this group is not based on the assumption that they are familiar with eating insects; rather, it is to explore how they approach the future of the food system (PFBEI) in a nature-based destination sensitive to the climate crisis, independent of direct consumption.

Since a sampling frame could not be established, convenience sampling was used. The value of 384 derived from Cochran’s [38] formula served only as a practical recruitment target. It does not confer a ±5% margin of error on this non-probability sample, and it is not a model-specific SEM power calculation. General sample-size rules and AVE values do not demonstrate adequate power for individual paths; requirements depend on model properties and parameter magnitudes [39]. The analysis is therefore based on the available 398 retained observations, without a claim of demonstrated power for every coefficient. An approximate 1:1 gender quota was applied during recruitment, yielding 199 women and 199 men in the analyzed sample. This balance does not eliminate selection bias or establish population representativeness. Data were collected between 22 July and 7 August 2026, via face-to-face interviews and tablets while participants were completing their day trips or resting during their visit. The study was reviewed by the Ethics Committee for Social and Human Sciences at Recep Tayyip Erdoğan University and was deemed ethically sound (Decision No. 2026/178).

### 4.2. Measurement Instruments and Reverse Coding

The survey form consists of two sections. The first section contains 25 statements measuring the constructs, while the second section includes demographic questions. The statements were measured using a 5-point Likert scale (1 = Strongly Disagree, 5 = Strongly Agree). The scales were prepared in Arabic and English; linguistic equivalence was confirmed through a back-translation process conducted by independent translators and expert academics.

- Climate Change Risk Perception (CCRP): Five statements adapted from the study by Zobeidi et al. [23] and measured using a five-point agreement scale were used.
- Perceived Future Benefits of Edible Insects (PFBEI): Measured using 4 statements adapted to insects by the researchers from the study by Weinrich et al. [25] (e.g., “It will be cheaper to meet global protein demand”).
- Memorable Tourism Experience (MTE): Five statements based on Zhang et al. [26] and Kim et al. [8] were administered during the visit, with the prefix “As a responsible tourist…”. They assess current evaluations of memorable experience qualities. The wording and timing were adaptations of the source measures; their potential framing effects and lack of post-visit memory assessment limit construct interpretation (Section 2.3).
- Place Attachment (PA): The 11 statements developed by Brown and Raymond [28] were adapted directly with reference to Ayder Plateau. To control for participants’ tendency to give careless or monotonous responses (acquiescence bias), items PA9 and PA10 in the place attachment dimension were formulated in a negative/reverse format. It was observed that item PA9 (“When I’m on vacation in Ayder Plateau, I’m not as satisfied as I am when I’m on vacation anywhere else”), whose negative comparative wording was difficult to interpret across language versions, created difficulties in comprehension. Before the dataset was analyzed, these two items were reverse-coded (1 = 5, 2 = 4, …, 5 = 1) to align them with the direction of the overall index.

### 4.3. Preliminary Analyses: Data Cleaning and Normality

A total of 435 survey forms were collected during the fieldwork. To identify multivariate outliers, the Mahalanobis distance (D^2^) was calculated for the 25 observed variables [40]. Thirty-seven questionnaires (8.5%) exceeded the χ^2^ threshold of 52.62 at *p* < 0.001 with 25 degrees of freedom and were excluded, leaving 398 observations. This criterion identifies unusual multivariate response profiles, not necessarily erroneous or careless responses. Given the ordinal item responses and the evidence of multivariate nonnormality, the χ^2^ cutoff should be treated as a screening rule rather than proof of invalid observations. The 37 excluded questionnaires were not retained in the stored dataset after screening, so a sensitivity analysis reinstating them could not be conducted. Because these cases represent 8.5% of the collected questionnaires, this is a serious limitation: the structural estimates reported in Section 5 may depend on the screening decision, and their validity for the full set of collected responses cannot be verified (Section 8).

The gender distribution of the sample is balanced (n = 199 women, n = 199 men). The age groups with the highest representation are 21–30 years (43.2%) and 31–40 years (20.4%). In terms of educational attainment, 33.7% of the participants hold a bachelor’s degree, and 32.7% are high school graduates. The item-level univariate skewness (−1.035 to −0.047) and kurtosis (−1.413 to 0.132) values (Table 1) fell within the thresholds recommended for structural equation modeling (|skewness| < 3, |kurtosis| < 10) [41] and all fall within the stricter ±2 range. In contrast, the Mardia multivariate kurtosis coefficient was calculated as 829.13 (the expected value under a normal distribution is 624) and the critical ratio as 57.92; since this value is significantly above the threshold of 5, the data are not multivariate normally distributed [42,43]. Multivariate skewness is also significant (χ^2^(2600) = 6552.03; *p* < 0.001). Therefore, the resampling bootstrap technique was preferred for testing indirect paths, and model fit was additionally assessed using the Bollen–Stine bootstrap test [44].

Within the retained dataset, 46 respondents (11.6%) had a score of 5 on all 25 items after reverse coding. This is not the same as selecting the highest response option for every original item: scores of 5 on the reverse-coded PA9 and PA10 correspond to original responses of 1. The pattern is therefore consistent with uniformly favorable answers but does not, by itself, establish indiscriminate agreement with all item wordings. Response-quality assessment benefits from multiple indicators rather than a single response pattern [45]. These observations were retained in the main analysis and excluded in a separate sensitivity model (N = 352; Section 5.4). This comparison addresses the influence of this particular pattern; it neither validates all retained responses nor evaluates the 37 excluded outliers.

## 5. Findings

### 5.1. Measurement Model (CFA), Dimensionality, and Validity

Confirmatory Factor Analysis (CFA) was conducted using AMOS 24 software to test the measurement model. The revised structural model, bootstrap, common-method variance, and sensitivity analyses were conducted in the R environment using the lavaan 0.6-17 package [46], employing Wishart likelihood compatible with AMOS; both software programs produced identical estimates for the measurement model. In the initial analysis, it was determined that the standardized load of the CCRP5 item, which assesses personal health risk, fell below the threshold value (λ = 0.42). In line with the theoretical framework of van der Linden [3] and Zobeidi et al. [23], which emphasizes that the perception of individual health threats is distinct from that of global/ecological threats, the CCRP5 item was excluded from the analysis to improve the measurement specification; its removal narrows CCRP to general and societal threats and does not itself establish validity.

To reflect the common expression effect of items PA9 and PA10, which were negatively worded and reverse-coded in the same direction, covariance was defined between the error terms of these two items. The four-factor measurement model demonstrated acceptable fit: χ^2^ = 574.00; df = 245; *χ*^2^/*df* = 2.343; CFI = 0.952; TLI = 0.946; RMSEA = 0.058 (90% CI [0.052; 0.064]); SRMR = 0.049. Standardized factor loadings, t-values, composite reliability (CR), average variance extracted (AVE), and Cronbach’s α values for the measurement model are presented in Table 2. To validate the structure of the Place Attachment (PA) scale, this model was compared to a two-dimensional model (based on the item order in the Brown and Raymond [28] scale) in which items PA1–PA6 represent place identity and items PA7–PA11 represent place dependence. The two-dimensional model (χ^2^ = 521.22; df = 241; *χ*^2^/*df* = 2.163; CFI = 0.959; TLI = 0.953; RMSEA = 0.054; SRMR = 0.043) demonstrated a statistically better fit (Δχ^2^(4) = 52.78; *p* < 0.001). However, the latent correlation between the two dimensions is 0.946, and even the lower limit of the confidence interval is above 0.90 (95% CI [0.924; 0.968]); this indicates that there is no discriminant validity between the dimensions. Therefore, in accordance with the principle of theoretical parsimony (simplicity), PA was conceptualized as a unidimensional structure in the structural model.

The threshold values for Composite Reliability (CR > 0.70) and Average Variance Extracted (AVE > 0.50) were exceeded for all constructs, and the reported indices supported convergent validity [47]. The relatively low factor loading of item PA9 (λ = 0.579) is consistent with the difficulty in understanding caused by the negative comparative wording in the translation process. Discriminant validity was confirmed using the Fornell–Larcker criterion (all AVE values are greater than the corresponding correlation squares) and HTMT ratios. All HTMT values are below the 0.85 threshold (Table 3).

Positive and statistically significant (*p* < 0.01) relationships were found among all variables. The strongest correlation was between PA and MTE (*r* = 0.706), while the weakest correlation was between PFBEI and MTE (*r* = 0.307) (Table 4).

### 5.2. Structural Equation Modeling (Direct Paths)

The covariance between the PFBEI and MTE residuals was freely estimated in the structural model [34]. Fixing this covariance to zero produced significantly poorer fit (Δχ^2^(1) = 12.98; *p* < 0.001); the estimated residual correlation was 0.209 (*p* < 0.001). The main model reported in Table 5 and Table 6 uses the freely estimated covariance. Its saturated structural part yields the same fit indices as the measurement model: χ^2^ = 574.00; df = 245; χ^2^/df = 2.343; CFI = 0.952; TLI = 0.946; RMSEA = 0.058; SRMR = 0.049; GFI = 0.890; AGFI = 0.866. Consequently, these fit statistics do not establish the direction of the structural relationships. The Bollen–Stine bootstrap test (2000 resamples) rejected exact fit (*p* < 0.001), indicating remaining model–data discrepancy; the approximate fit indices do not negate that result. The proportions of variance accounted for by the specified regressions were R^2^(PFBEI) = 0.197, R^2^(MTE) = 0.135, and R^2^(PA) = 0.609.

Direct and indirect coefficients are interpreted as cross-sectional associations conditional on the specified model [36]. Their signs, significance, and relative magnitudes do not identify temporal ordering or rule out reverse relationships and omitted common causes. The hypotheses are not presented as causal confirmations. Table 5 describes the main model, and its last column summarizes the result of each hypothesis under the method-factor specification so that the robustness of each association can be read directly, while Table 7 is essential to the interpretation of its sensitivity to method-factor adjustment.

**Table 5:** Direct Path Coefficients in the Structural Model.

Hyp.	Path/Relationship	Standardized Coefficient (β)	t-Value	*p*-Value	Main-Model Result	With Method Factor (Table 7)
H1	CCRP → MTE	0.367	6.118	<0.001	Supported	Supported
H2	CCRP → PFBEI	0.444	7.522	<0.001	Supported	Supported
H3	CCRP → PA	0.023	0.487	0.626	Not supported	Not supported
H4	PFBEI → PA	0.165	3.731	<0.001	Supported	Not supported (β = 0.022; *p* = 0.750)
H5	MTE → PA	0.699	12.611	<0.001	Supported	Supported

Note: The t-values correspond to unstandardized estimates; β values are standardized. The PFBEI–MTE residual covariance was freely estimated. Significance in this table refers to the main model only. H4 becomes nonsignificant in the ULMC specification (Table 7). The last column reports each result with the unmeasured latent method factor.

**Table 6:** Bootstrap Analyses of Parallel Indirect Paths.

Hyp.	Path/Relationship	Coefficient (β)	Bootstrap SE	95% Confidence Interval (CI)	*p*-Value	Association Pattern	Main-Model Result
H6	CCRP → PFBEI → PA	0.073	0.024	[0.034; 0.130]	<0.001	Indirect association	Supported in main model; not robust to method factor
H7	CCRP → MTE → PA	0.257	0.047	[0.172; 0.353]	<0.001	Indirect association	Supported
-	**Total indirect association**	0.330	0.053	[0.233; 0.443]	<0.001	-	-
-	**Direct coefficient** (CCRP → PA)	0.023	0.049	[−0.072; 0.118]	0.654	Nonsignificant direct coefficient	-
-	**Total association** (*c*)	0.353	0.061	[0.230; 0.466]	<0.001	-	-
-	**Difference** (H7–H6)	0.183	0.051	[0.088; 0.286]	<0.001	-	-

Note: Standardized estimates; 5000-resample bias-corrected (BC) bootstrap. These are model-dependent indirect associations, not evidence of causal mediation. Main-model significance must be read alongside Table 7: H6 is nonsignificant with the method factor, whereas H7 remains significant. The direct-path *p*-value here is based on the bootstrap procedure; Table 5 reports the model-based test.

**Table 7:** Sensitivity Analyses of the Common Method Variance and Response Pattern.

Path	β (Main Model)	β (ULMC)	*p* (ULMC)	β (N = 352)	*p* (N = 352)
H1: CCRP → MTE	0.367	0.269	<0.001	0.195	0.002
H2: CCRP → PFBEI	0.444	0.311	<0.001	0.205	0.001
H3: CCRP → PA	0.023	0.007	0.888	0.017	0.732
H4: PFBEI → PA	0.165	0.022	0.750	0.146	0.001
H5: MTE → PA	0.699	0.710	<0.001	0.694	<0.001
H6: CCRP → PFBEI → PA	0.073	0.007	0.755	0.030	0.022
H7: CCRP → MTE → PA	0.257	0.191	0.001	0.136	0.002

Note. ULMC: unmeasured latent method factor loading on all items and specified as orthogonal to the substantive factors. N = 352: the 398-case dataset after excluding 46 respondents with all scores equal to 5 following reverse coding. *p*-values in the sensitivity models use the delta method. The two sensitivity specifications are separate; neither reinstates the 37 excluded outliers.

### 5.3. Parallel Indirect Paths

To estimate the specific indirect associations of climate change risk perception with place attachment (H6 and H7), the simultaneous model included both PFBEI and MTE [34]. Each indirect coefficient is the product of the two corresponding regression coefficients. These products and their contrast were evaluated using bias-corrected (BC) bootstrap confidence intervals based on 5000 resamples. Resampling quantifies uncertainty under the fitted model; it does not supply temporal ordering or remove confounding.

In the main model, the specific indirect coefficients via PFBEI (β = 0.073; 95% CI [0.034; 0.130]) and MTE (β = 0.257; 95% CI [0.172; 0.353]) were positive, and their confidence intervals excluded zero. The adjusted CCRP–PA coefficient was not significant (β = 0.023; 95% CI [−0.072; 0.118]); this is insufficient evidence of a conditional association, not proof that the population coefficient is zero. The estimated contrast favored the MTE coefficient (difference = 0.183; 95% CI [0.088; 0.286]). Although such a significance pattern is sometimes termed “indirect-only mediation” [35], that causal-sounding classification is not used as a substantive conclusion here. The PFBEI indirect association did not persist with the method factor, as reported below.

### 5.4. Common Method Variance and Sensitivity Analyses

Since all data were collected from a single source using a single form, common method variance was tested in two ways [48]. In Harman’s one-factor test, the first component in the unrotated principal component analysis of the 24 items explained 44.45% of the total variance, and four components with eigenvalues greater than 1 were obtained. The confirmatory model, in which all items loaded on a single factor, showed very poor fit (χ^2^ = 2350.29; df = 251; CFI = 0.691; RMSEA = 0.145; Δχ^2^(6) = 1776.29; *p* < 0.001). Neither the percentage explained by the first component nor poor single-factor fit rules out common method bias. Therefore, an unmeasured latent method factor (ULMC), defined as unrelated to the structures and loaded on all items, was added to the model. The method factor significantly improved the fit (χ^2^ = 424.09; df = 221; CFI = 0.970; RMSEA = 0.048; SRMR = 0.028; Δχ^2^(24) = 149.91; *p* < 0.001), and all method loadings were found to be significant (ranging from 0.220 to 0.736). Under this specification, the method factor accounted for an average of 19.2% of item variance, compared with 45.8% for the substantive factors. The 19.2% is a model-dependent variance estimate, not the percentage bias in the structural coefficients. The effect of the method factor is particularly evident in the reverse-scored items PA9 and PA10, in PA11, and in the PFBEI items.

With the method factor included (Table 7), H1, H2, H5, and H7 remained significant. In particular, CCRP → PFBEI remained significant (H2; β = 0.311; *p* < 0.001). In contrast, PFBEI → PA (H4; β = 0.022; *p* = 0.750) and the specific indirect association through PFBEI (H6; β = 0.007; *p* = 0.755) became nonsignificant. Thus, the sensitivity concerns the PFBEI pathway to attachment, not every association involving PFBEI. H4 and H6 are therefore supported in the main model but not robust to method-factor adjustment. The ULMC model is a sensitivity analysis: its orthogonality and loading assumptions do not guarantee complete removal of method bias or distinguish method variance from all shared substantive variance [48]. Persistence of the MTE pathway under this specification therefore does not establish that it is unbiased.

When the 46 respondents with uniformly maximum recoded scores were excluded (N = 352), the significance decisions in the main model were unchanged, but several estimates were smaller. CCRP → MTE declined from 0.367 to 0.195, CCRP → PFBEI from 0.444 to 0.205, and the specific indirect association via MTE from 0.257 to 0.136 (Table 7). These are descriptive changes between estimates, not a formal statistical test of coefficient differences. The comparison concerns a subset of the 398 retained cases only. No model using all 435 questionnaires was estimated, so this sensitivity check cannot establish that excluding the 37 Mahalanobis outliers was inconsequential. The sensitivity of the results to excluding the 37 Mahalanobis outliers therefore remains a serious, unresolved limitation.

## 6. Discussion

This study examined cross-sectional associations between general climate risk perception and place attachment in a convenience sample of Arabic-speaking visitors to Ayder Plateau. Current evaluations of memorable experience qualities had the strongest association with attachment. Although both specific indirect coefficients were significant in the main model, only the MTE indirect association remained significant with the latent method factor. The findings therefore do not support two equally robust pathways to attachment.

All results must be interpreted with caution because several variables that could account for the observed relationships were not measured. General environmental orientation could underlie climate risk perception, food-benefit expectations, environmentally framed experience evaluations, and attachment to a protected natural destination simultaneously; had it been measured, the association between PFBEI and place attachment might have disappeared even in the main model. Prior visitation and length of stay could account for both more favorable experience evaluations and stronger attachment, and therefore for part or all of the MTE–PA association. Food neophobia, disgust sensitivity, and halal perceptions are established correlates of edible-insect evaluations and may confound the associations involving PFBEI. Because none of these variables was measured, the present data cannot exclude the possibility that they fully account for the reported relationships. This is a serious limitation that applies to every structural association discussed below.

### 6.1. The Role of Experience and Alternative Food Expectations (H1 and H2)

The positive CCRP–MTE association (H1; β = 0.367) is compatible with the interpretation that visitors who report greater climate concern also value their current nature-based experience more positively [8,32]. However, destination-specific threat appraisal, perceived rarity, and memory encoding were not measured, so the last-chance tourism account remains an interpretive possibility rather than a demonstrated mechanism. A positive experience may also heighten environmental concern. In addition, the “As a responsible tourist…” prefix could encourage environmental self-presentation and inflate the association. Its contribution cannot be isolated by the present questionnaire or ruled out by the ULMC result. This finding concerns in-visit ratings, not memories shown to persist after departure.

The positive relationship between CCRP and PFBEI (H2; β = 0.444) is consistent with established cross-sectional studies reporting that individuals who perceive climate risk as higher evaluate alternative proteins more positively [17]. However, the construct measured here is not consumption intention but rather the expectation of benefits/solutions regarding the food crisis. Indeed, Onat et al. [16] demonstrated that threat perception is distinct from intention. In this sample, higher CCRP scores covaried with stronger expected food benefits. Although this is conceptually related to response efficacy [4], it does not demonstrate a measured efficacy mechanism or willingness to consume insects.

### 6.2. Interpreting the Nonsupport of H3: Direct and Total Associations

H3 was not supported, but this result should be interpreted through the distinction between the total and the direct association. Climate risk perception and place attachment were positively correlated (*r* = 0.336, *p* < 0.01; Table 4), and the total association estimated in the model was positive and significant (β = 0.353; 95% CI [0.230; 0.466]; Table 6). What was nonsignificant was the direct coefficient that remained once MTE and PFBEI were included (β = 0.023; *p* = 0.626; Table 5). In statistical terms, most of the CCRP–PA association was accounted for by the two indirect associations, particularly the one through MTE (β = 0.257). Because the data are cross-sectional, this decomposition describes how the association is distributed within the specified model; it is not evidence that MTE or PFBEI causally transmit climate risk perception into attachment [36].

A substantive interpretation concerns the different referents of the two constructs. After the removal of the personal-health item, CCRP captures a general judgment about global and societal climate threats (Section 2.1), whereas PA is directed at Ayder Plateau. Scannell and Gifford [27] emphasize that attachment is always directed toward a specific place, and research on place-related risk shows that the relationship between risk perception and attachment is neither direct nor automatic, even when the risk concerns the place itself [12,13]. In the present model, MTE and PA share the same destination as their referent, which may help explain why the experience evaluations, rather than the general threat judgment, carried most of the association with attachment. Construal level theory offers a complementary possibility: objects and events that are psychologically distant tend to be represented more abstractly [49], and climate change can be perceived as psychologically distant on some dimensions [50]. A general climate threat judgment may therefore be represented at a more abstract level than the concrete bond with a particular destination and relate to it mainly through more concrete, place-specific evaluations. Psychological distance was not measured, so this account is a possible explanation rather than a tested mechanism.

These possible explanations can be related to the setting only with caution. Descriptive sources characterize Ayder as a highland destination within Kaçkar Mountains National Park, known for its cool climate, greenery, and hot springs, and the Eastern Black Sea Region as a summer destination for visitors from Gulf and Middle Eastern countries [18]. Such sources describe the destination and the regional market; they do not show why the present respondents chose Ayder, how they perceived its climate, or whether they regarded it as threatened, and none of these was measured. Theoretical considerations nevertheless suggest why a direct association need not be expected. The place dimension of attachment includes the physical characteristics of a setting [27], and place dependence reflects the extent to which a place meets visitors’ functional needs [28]; attachment grounded in such attributes would not necessarily vary with a general climate threat judgment. In addition, research linking memorable experiences with attachment among tourists in short-term accommodation [11] suggests that attachment can develop through the experience of the visit itself, which is consistent with the larger role of the experience evaluations in the present model. Whether these considerations apply to the present respondents is unknown, because visit motivation, destination-specific threat perception, psychological distance, length of stay, and prior visitation were not measured (Section 8). They are therefore offered as theoretically grounded possible explanations of the nonsignificant direct coefficient, not as its causes, and they cannot be distinguished from confounding or alternative structural orderings.

### 6.3. Associations with Place Attachment and Sensitivity to Method Variance (H4 and H5)

In-visit evaluations of memorable tourism experience qualities had the strongest standardized association with place attachment (H5; β = 0.699). What was measured, however, is a momentary evaluation of the ongoing experience, not a memorable tourism experience in the sense used in the MTE literature, where the construct refers to experiences recalled after travel [8]. Although the pattern resembles associations reported in retrospective MTE research [9,11], the present finding cannot be taken as confirming or extending that literature, and it cannot be generalized to MTE as a retrospective construct. Whether these evaluations persist as memories, and whether recalled experiences would show the same association with attachment, was not examined. The coefficient remained substantial with the method factor (β = 0.710). These results are compatible with an experience–attachment relationship but also with reverse appraisal, whereby tourists already attached to Ayder evaluate their visit more positively. Neither the cross-sectional model nor its method-factor adjustment can discriminate between these explanations.

The main-model PFBEI–PA coefficient (H4; β = 0.165) was considerably smaller than the MTE–PA coefficient (β = 0.699). This difference is plausible because PFBEI, unlike MTE, does not refer to Ayder itself. One possible explanation for the positive association is value congruence (Section 2.5): expectations about sustainable food solutions may reflect environmental values, and such values may be associated with attachment to a protected natural destination. Biospheric values were not measured, however, so this explanation was not tested; the association also did not depend on insect consumption, which was not part of the experience studied. With the latent method factor, the coefficient was near zero and nonsignificant (β = 0.022; *p* = 0.750), so H4 was not supported in that specification. This result does not prove that all of the initial association was a method artifact. The PFBEI–PA association may also reflect general environmental orientation rather than a relationship between food-benefit expectations and attachment, and this possibility could not be tested.

### 6.4. Interpretation of the Specific Indirect Associations (H6 and H7)

The indirect association through MTE was larger than that through PFBEI in the main model (difference = 0.183; 95% CI [0.088; 0.286]) and remained significant in the ULMC model (β = 0.191; *p* = 0.001). The PFBEI indirect association became nonsignificant (β = 0.007; *p* = 0.755). This contrast favors the place-specific experience association within the specifications examined, not a causal claim that experience transmits climate risk perception into attachment. Excluding the 46 respondents with uniformly maximum recoded scores also reduced the MTE indirect coefficient to 0.136 and the PFBEI indirect coefficient to 0.030. Thus, persistence of a significance decision should not be confused with stability of magnitude. Robustness to reinstating the 37 outliers remains untested and is a serious limitation (Section 8).

### 6.5. Sample Heterogeneity and Cultural Variables

PFBEI averaged 3.10 on the five-point scale (SD = 1.31), close to the scale midpoint with substantial dispersion. Because the national composition of the sample is unknown, this dispersion cannot be attributed to, or separated from, national, cultural, or religious differences, and the study cannot determine whether such differences in edible-insect perceptions or place attachment exist. Studies in Saudi Arabia report heterogeneous responses and barriers related to hygiene, sensory qualities, and halal compliance [21,22], but whether comparable patterns characterize the present sample cannot be assessed. Pooled estimates may therefore conceal differences between national groups. The absence of country data is a design flaw that precludes precise cultural interpretation of the findings.

## 7. Conclusions and Practical Implications

In this sample of Arabic-speaking visitors to Ayder Plateau, climate risk perception was positively associated with in-visit evaluations of memorable experience qualities and with beliefs about the future benefits of edible insects, and both were associated with place attachment. The two pathways, however, differed in robustness. The indirect association through experience evaluations remained significant with a latent method factor and after excluding uniformly maximum responses, whereas the PFBEI pathway to attachment was supported in the main model but did not persist with the method factor. The contribution is therefore a context-specific account of two routes linking climate concern and attachment to a nature-based destination: a robust place-based route through visitors’ evaluations of their ongoing experience, and a value-based route through edible-insect benefit expectations that requires confirmation with measured values and separated measurement occasions.

These conclusions are conditional on the screened sample of 398 respondents. Because the 37 excluded questionnaires could not be reinstated, whether the estimates are sensitive to this exclusion is unknown, and this limits the validity of all reported structural results. The experience finding concerns a momentary evaluation of the ongoing experience. It cannot be generalized to memorable tourism experience as a retrospective, post-travel construct, and it should not be read as evidence about the relationship between remembered experiences and place attachment. In addition, the cross-sectional design, the unrecorded country composition, and the unmeasured covariates discussed in Section 6 mean that the results establish associations rather than causal mediation and should be interpreted with caution (Section 8).

For destination managers, the association between current experience evaluations and attachment supports further investigation of experience quality in nature-based settings. Nature interpretation and visitor education about conservation are plausible practices to evaluate, but this study did not test whether they increase attachment or whether climate-focused messages improve experiences. No recommendation to introduce or promote edible insects at Ayder follows from these results. Practical interventions should be assessed independently, ideally with pre-visit and post-visit measurements and comparison conditions.

## 8. Limitations and Future Research

The theoretical implications of this study should be evaluated in light of several important limitations:

1. Data screening and untested robustness, a serious limitation: Thirty-seven of the 435 collected questionnaires (8.5%) were excluded as multivariate outliers, and these cases were not retained in the stored dataset after screening. A sensitivity analysis reinstating them was therefore not possible. This is a serious limitation that affects the validity of the reported results, not a minor reporting issue. Exclusions of this magnitude can alter the covariance structure on which all structural coefficients and indirect associations depend, and statistical atypicality does not by itself establish that the excluded responses were invalid, particularly with nonnormal ordinal indicators. The direction and size of any change in the estimates are unknown. The N = 352 analysis examines only the influence of uniformly favorable recoded responses within the retained sample and cannot substitute for the missing analysis. The present findings should therefore be regarded as provisional until replicated. Future studies should retain all collected responses, specify screening rules in advance, and report results both with and without excluded cases, together with estimation methods that are robust to nonnormality and atypical observations.
2. Questionnaire design flaw, unrecorded country composition: Country of origin and country of residence were not recorded, although the sample was recruited as Arabic-speaking tourists from different countries. This omission is a flaw in the questionnaire design, not a matter that can be addressed by moderating generalizability claims. The structural estimates are pooled across an unknown mix of national groups, so cultural differences in edible-insect perceptions, place attachment, or the relationships among the constructs may have been overlooked. Measurement equivalence across national groups could not be tested, and the findings cannot support country-specific or precise cultural interpretations. Future research with Arabic-speaking tourists should record country of origin and residence as standard demographic items, recruit sufficiently large national subgroups, and test measurement invariance before comparing structural relationships across groups.
3. Unmeasured covariates, a serious limitation: General environmental identity or biophilic orientation, food neophobia, halal perceptions, disgust sensitivity, prior visitation, and length of stay were not measured. These variables could partly or fully account for the observed relationships, including the PFBEI–PA and MTE–PA associations. Age, gender, education, and survey language were not included as covariates in the structural model. The reported paths therefore cannot be interpreted as independent of these possible confounders. Their absence cannot be remedied by a theoretical explanation or the latent method factor. Future studies should specify and measure substantively justified covariates and assess language and country measurement equivalence before drawing comparative cultural conclusions.
4. Cross-sectional measurement and causal direction: All constructs were measured from the same respondents at one occasion during their visit. The indirect coefficients are products within a specified covariance model, not evidence of temporal mediation [36]. Existing attachment may influence experience evaluations, and a positive nature-based experience may influence climate concern. Unmeasured common causes could also account for their covariation. The saturated structural component cannot establish that the proposed ordering is superior to plausible reverse orderings. Longitudinal measurement before, during, and after travel, together with experimental tests of specific interventions, is needed to evaluate these alternatives.
5. Measurement, framing, and common method variance: MTE represents a momentary evaluation of the ongoing experience, measured during the visit; it is not equivalent to the retrospective construct examined in the MTE literature, and the MTE findings cannot be generalized to that construct. The “As a responsible tourist…” prefix may have primed an environmental identity or socially desirable responses. Neither acceptable measurement-model fit nor the ULMC analysis establishes equivalence with an unframed, retrospective MTE measure. The reverse-worded PA items also warrant caution in translated questionnaires. In the ULMC specification, the method factor accounted for an average of 19.2% of item variance, and the PFBEI pathway to attachment was nonsignificant. This pattern limits substantive interpretation and cannot be dismissed because Harman’s test yielded less than 50% on the first component. A general method factor cannot identify the specific bias source or guarantee unbiased remaining coefficients. Future research should separate measurement occasions, compare framed and unframed items, assess post-visit recall, and include appropriate marker and response-quality indicators.
6. Sampling and generalizability: Convenience recruitment at one destination over 17 summer days provides no probability-based margin of error or statistical representation of Arab tourists. The gender quota does not correct other selection processes, and a model-specific power analysis was not conducted. A local news report described reduced regional arrivals around the fieldwork period [20], but the study lacks comparative visitor data to determine the extent or direction of any compositional change. The findings cannot establish typical-season patterns, country-specific relationships, or cross-cultural equivalence. Replication across destinations, seasons, and clearly reported country groups is required.

## Figures and Tables

**Table 1:** Skewness and Kurtosis Values for Scale Items.

Variables and Expressions	Skewness	Kurtosis
CCRP Expressions		
CCRP1	−0.501	−0.793
CCRP2	−0.648	−0.553
CCRP3	−0.717	−0.382
CCRP4	−0.456	−0.866
CCRP5	−0.199	−1.256
PA Expressions (Ayder Plateau Reference)		
PA1	−0.912	−0.487
PA2	−0.945	−0.335
PA3	−0.959	−0.259
PA4	−0.761	−0.702
PA5	−1.013	−0.158
PA6	−0.873	−0.409
PA7	−0.968	−0.159
PA8	−0.795	−0.563
PA9 (Reverse-Coded)	−0.563	−1.009
PA10 (Reverse-Coded)	−0.699	−0.690
PA11	−0.709	−0.595
PFBEI Expressions		
PFBEI1	−0.166	−1.310
PFBEI2	−0.047	−1.320
PFBEI3	−0.104	−1.413
PFBEI4	−0.060	−1.375
MTE Statements (preceded by “As a responsible tourist…”)		
MTE1	−1.031	0.123
MTE2	−0.888	−0.164
MTE3	−0.944	−0.132
MTE4	−0.960	−0.039
MTE5	−1.035	0.132

**Table 2:** Findings of Confirmatory Factor Analysis and Reliability Analysis.

Dimensions and Items	Standard Load (λ)	t Value	CR	AVE	Cronbach’s α
CCRP (Climate Risk Perception)			0.802	0.506	0.798
CCRP1	0.763	Constant			
CCRP2	0.778	13.607			
CCRP3	0.674	12.167			
CCRP4	0.620	11.242			
PA (Place Attachment)			0.949	0.633	0.949
PA1	0.812	16.857			
PA2	0.852	17.800			
PA3	0.867	18.155			
PA4	0.863	18.068			
PA5	0.789	16.327			
PA6	0.841	17.548			
PA7	0.861	18.029			
PA8	0.782	16.149			
PA9 (Reverse-Coded)	0.579	11.636			
PA10 (Reverse-Coded)	0.720	14.739			
PA11	0.743	Constant			
PFBEI (Perceived Future Benefits of Edible Insects)			0.917	0.734	0.917
PFBEI1	0.854	21.031			
PFBEI2	0.867	21.513			
PFBEI3	0.864	21.390			
PFBEI4	0.844	Constant			
MTE (Memorable Tourism Experience)			0.891	0.621	0.890
MTE1	0.795	Constant			
MTE2	0.836	18.298			
MTE3	0.822	17.920			
MTE4	0.715	15.066			
MTE5	0.767	16.424			

**Table 3:** Discriminant Validity: HTMT Ratios.

Structures	1. CCRP	2. PA	3. PFBEI	4. MTE
1. CCRP	-			
2. PA	0.384	-		
3. PFBEI	0.462	0.447	-	
4. MTE	0.370	0.768	0.339	-

**Table 4:** Correlation Coefficients, Means, and Standard Deviations for Variables.

Scales	Mean	SD	1	2	3	4
1. CCRP	3.622	0.992	1			
2. PA	3.815	1.082	0.336 **	1		
3. PFBEI	3.100	1.312	0.396 **	0.419 **	1	
4. MTE	3.970	0.990	0.312 **	0.706 **	0.307 **	1

Note: ** *p* < 0.01.

## Data Availability

The data supporting the findings of this study are not publicly available due to privacy and ethical restrictions but are available from the corresponding author upon reasonable request.

## Abbreviations

The following abbreviations are used in this manuscript:

AGFI	Adjusted Goodness-of-Fit Index
AMOS	Analysis of Moment Structures
AVE	Average Variance Extracted
BC	Bias-Corrected
CCRP	Climate Change Risk Perception
CFA	Confirmatory Factor Analysis
CFI	Comparative Fit Index
CI	Confidence Interval
CR	Composite Reliability
df	Degrees of Freedom
GFI	Goodness-of-Fit Index
HTMT	Heterotrait–Monotrait Ratio
MTE	Memorable Tourism Experience (in-visit evaluation)
PA	Place Attachment
PFBEI	Perceived Future Benefits of Edible Insects
RMSEA	Root Mean Square Error of Approximation
SD	Standard Deviation
SE	Standard Error
SRMR	Standardized Root Mean Square Residual
TLI	Tucker–Lewis Index
ULMC	Unmeasured Latent Method Factor
